# Deficiency of Interleukin-1 Receptor Antagonist: A Case with Late Onset Severe Inflammatory Arthritis, Nail Psoriasis with Onychomycosis and Well Responsive to Adalimumab Therapy

**DOI:** 10.1155/2019/1902817

**Published:** 2019-08-04

**Authors:** Necil Kutukculer, Anne Puel, Sanem Eren Akarcan, Kunihiko Moriya, Neslihan Edeer Karaca, Melanie Migaud, Jean-Laurent Casanova, Guzide Aksu

**Affiliations:** ^1^Ege University Faculty of Medicine, Department of Pediatric Immunology and Rheumatology, Izmir, Turkey; ^2^Laboratory of Human Genetics of Infectious Diseases, Necker Branch, INSERM UMR 1163, Paris, EU, France; ^3^Paris Descartes University, Imagine Institute, Paris, EU, France; ^4^St. Giles Laboratory of Human Genetics of Infectious Diseases, Rockefeller Branch, The Rockefeller University, New York, NY, USA; ^5^Howard Hughes Medical Institute, New York, NY, USA

## Abstract

DIRA (deficiency of the IL-1Ra) is a rare condition that usually presents in the neonatal period. Patients with DIRA present with systemic inflammation, respiratory distress, joint swelling, pustular rash, multifocal osteomyelitis, and periostitis. Previously, we reported a patient with a novel mutation in* IL1RN* with a healthy neonatal period, a late-onset of pustular dermatosis, inflammatory arthritis, and excellent response to canakinumab treatment. Herein, we are presenting a new case of late-onset DIRA syndrome, carrying a different mutation and showing different clinical findings. This patient is the first one in the literature with the inflammatory arthritis, nail psoriasis, and onychomycosis and with her remarkable response to monoclonal antibodies. The case responded well and fully recovered after treatment with adalimumab, but not with canakinumab. The DIRA disease can lead to death from multiple organ failures and if recognized early, the treatment with replacement of the deficient protein with biologic agents induces rapid and complete remission. Therefore, clinical symptoms should be learned exactly by the pediatricians, pediatric rheumatologists, and immunologists; and molecular analysis targeting this defect must be performed as early as possible.

## 1. Introduction

Autoinflammatory diseases are a group of disorders characterized by systemic inflammation without high-titer autoantibodies or autoantigen-specific T cells [1, 2]. Autoinflammatory diseases are clinically characterized by recurrent or persistent systemic inflammation, such as fever and organ-specific manifestations, such as rashes and osteoarticular, serosal, neurologic, or ocular manifestations [1–3].

These diseases are caused by dysregulated activation of the inflammasome, which is critical for the activation of the proinflammatory cytokine interleukin- (IL-) 1*β*, a powerful mediator of inflammatory responses [4]. The IL-1 receptor antagonist (IL1Ra) inhibits the activities of IL-1*α* and IL-1*β*. Recently, a severe autoinflammatory condition was found to be caused by biallelic mutations in* IL1RN* and named as deficiency of the IL-1Ra (DIRA) [5, 6]. Mutations in* IL1RN* lead to partial or complete absence of the IL-1Ra protein causing uncontrolled activity of IL-1 *α* and IL-1*β* on the IL-1Rs. The disease therefore results from an inability to downregulate the IL-1 response, and the resulting severe inflammatory reaction observed in these patients can resemble an acute severe systemic infection.

The DIRA syndrome is a rare condition characterized by perinatal-onset pustular dermatitis, multifocal aseptic osteomyelitis, periostitis, leukocytosis, joint swelling, systemic inflammation, and marked elevation in the levels of acute-phase reactants.

In 2015, we reported a 12-year-old girl with a novel mutation in* IL1RN *with a late-onset presentation in comparison to previously reported children with DIRA [7]. This case was the first reported patient with DIRA with an excellent response to canakinumab (recombinant human anti-human IL-1beta monoclonal antibody) treatment [7].

Herein, we are presenting a new case of DIRA syndrome, coming from the same small town as our previously identified patient, but carrying a different mutation. The case responded well and fully recovered after treatment with the monoclonal antibody adalimumab, but not with canakinumab.

## 2. Case Report

A 24-year-old girl, born to 4th degree consanguineous parents of Turkish origin, was admitted to our hospital with complaints of pain, swelling, and limited movement of left elbow, ankles, and both knees since the age of 11 years. Following a diagnosis of juvenile idiopathic arthritis, treatment with corticosteroids, methotrexate, and sulfasalazine was given at public hospital. As she was not responding to these treatments, she was hospitalized for further examination and therapy at our University Hospital Pediatric Rheumatology Clinic 12 years ago.

Her developmental milestones were normal for age. She had cataract surgery at the age of ten. She had been diagnosed with bilaterally hand and foot onychomycosis for at least five years and received antifungal therapies with no improvement although any fungus could be identified in cultures. Her sister had been diagnosed with juvenile idiopathic arthritis at seven years of age and deceased due to chronic renal failure at the age of 13.

**Figure 1:**
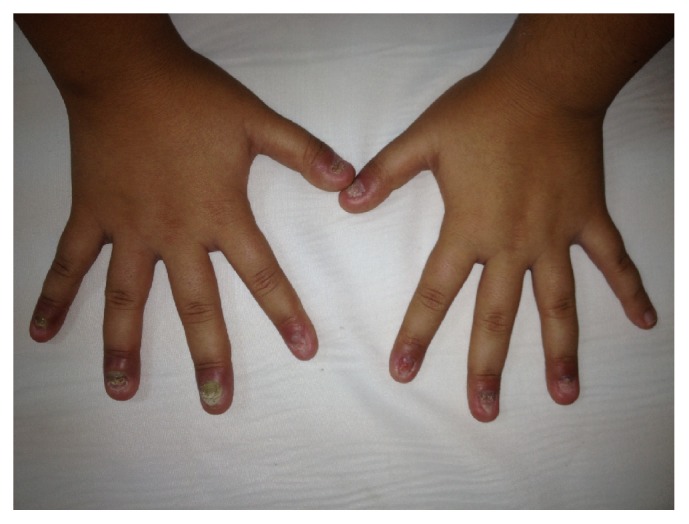
Dystrophic nails of the hand with nail psoriasis (leukonychia, nail plate crumbling, and onycholysis) accompanied by onychomycosis at admission.

On her first physical examination [at first admission, her weight was 24 kg (<3 percentile) and height 132 cm (<3 percentile)] she had failure to thrive. There was limitation in left elbow dorsiflexion and in hip abduction and swelling in both knees. There was also heel pain with tenderness of achilles tendon. Besides articular findings, the most important symptoms were dystrophic nails of hand and feet with onychomycosis. Dermatologic consultation revealed that she had nail psoriasis accompanied by onychomycosis (Figure 1). According to the consultation report, there were lesions in the nail matrix (leukonychia and nail plate crumbling) and separation of the nail plate from underlying nail bed (onycholysis). Her nails were painful and were causing restrictions in her daily activities.

Laboratory examinations were as follows: white blood cells 7960/mm3 (polymorphonuclear leukocytes 56%, lymphocytes 44%), hemoglobin 10.6 g/dl, hematocrit 32.8%, platelets 392000/mm3, C-reactive protein 5 mg/dl (normal <0.5 mg/dl), erythrocyte sedimentation rate 105 mm/hr (normal <15 mm/hr), and normal renal and liver function tests. Serum immunoglobulins (Ig) were very high as compared to age matched controls (IgG 2230 mg/dl, IgM 242 mg/dl, IgA 201 mg/dl) revealing hypergammaglobulinemia [8]. The search for anti-nuclear antibodies and rheumatoid factor was negative. Abdominal and renal ultrasonography were normal. Some of the microbiologic tests were as follows: anti-HBs (-), HbsAg(-), anti-HIV (-), Brucella agglutination test (-), CMV IgM (-), and IgG(+). Total IgE levels, eosinophil counts, absolute counts, and percentages of lymphocyte subsets and the oxidative burst activity of granulocytes were normal. She was evaluated for tuberculosis and was found to have two BCG (Bacillus Calmette-Guerin) scars, a negative tuberculin response, and a negative QuantiFERON assay result (Qiagen, Chadstone, Australia).

At the beginning of the long term follow-up (totally 12 years) she was diagnosed with refractory polyarticular juvenile idiopathic arthritis and nail psoriasis with onychomycosis. She was started on treatment with etanercept (Enbrel), methotrexate, and itraconazole with good response for a while for arthritic problems, but not for dermatologic disorders. Then, because of inadequate response, prednisolone at a dose of 1 mg/kg/day was added and also resulted in substantial improvement. She had experienced recurrent urinary tract infections and* Klebsiella pneumonia* was isolated from urine. Scintigraphic examinations, voiding cystography, and ultrasonography for kidneys were normal. Urine acid-fast bacilli test was negative. She did not have recurrent sinopulmonary infections.

Then,* Candida albicans* was isolated from swab material of nail. She was given local and systemic antifungal medications for onychomycosis with no response. As she had nail psoriasis with refractory onychomycosis, molecular genetic analyses for chronic mucocutaneous candidiasis and APECED syndrome were performed.* CARD9*,* AIRE,* and* STAT1 *genes were found to be wild-type.

Etanercept, methotrexate, and sulfasalazine therapies for about seven to eight years were successful for her arthritis, but she had no improvement for her nail psoriasis with onychomycosis. In addition, she developed a very severe arthritis in her coxofemoral joint when she was 20 years old. MRI of pelvis showed severe chronic inflammatory changes with sacroiliitis and ankylosis. Whole exome sequencing was performed and we identified a homozygous premature stop codon mutation c.85C>T(p.Arg29Ter) in* IL1RN *gene and was confirmed by Sanger sequencing. Both parents were heterozygous for the mutation. This novel mutation of* IL1RN* is predicted to produce a truncated protein that cannot bind the IL-1 receptor and is thus loss-of-function. Based on the clinical similarities with other DIRA patients described in the literature, having refractory chronic arthritis and psoriasis, this novel deleterious mutation is probably responsible for the patient phenotype.

**Figure 2:**
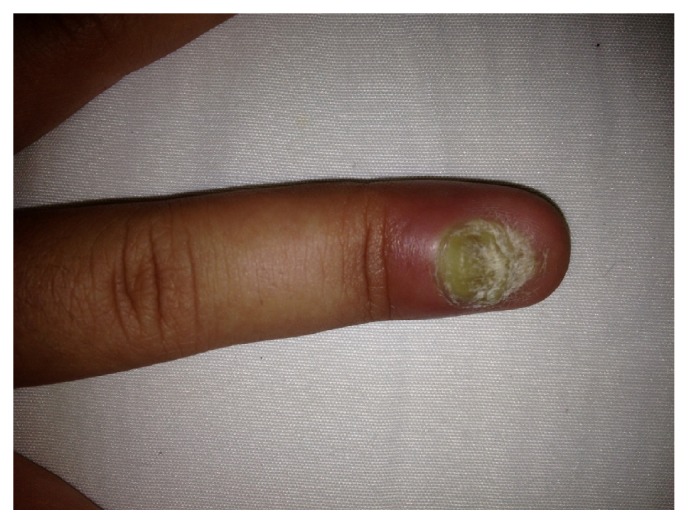
Improved nail psoriasis with onychomycosis and severe paronychia that persisted although the patient received nine months of canakinumab therapy.

**Figure 3:**
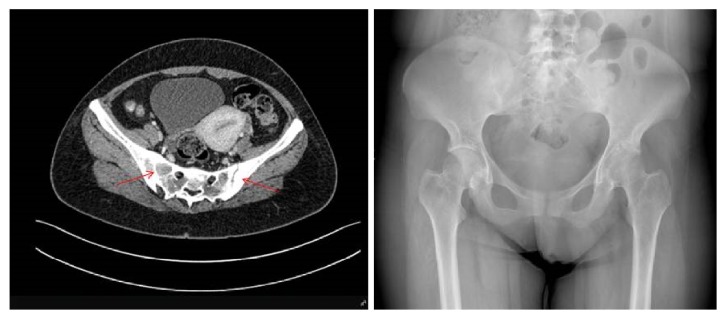
MRI and X-ray of sacroiliac joints showing total ankylosis of the right sacroiliac joint and partial ankylosis with irregularity on the nonsclerotic segments of the left sacroiliac joint (grade 3-4 sacroiliitis).

Treatment with canakinumab 150 mg subcutaneously once every 4 weeks was given for 9 months. Her arthritis features slightly recovered; however nail disease did not resolve but slightly improved (Figure 2). In addition, acute phase reactants did not decrease to normal levels. Both X-ray and new MRI showed chondrolysis in left hip joint as well as sacroiliitis (grade III-IV) and ankylosis in some areas (Figure 3).

**Figure 4:**
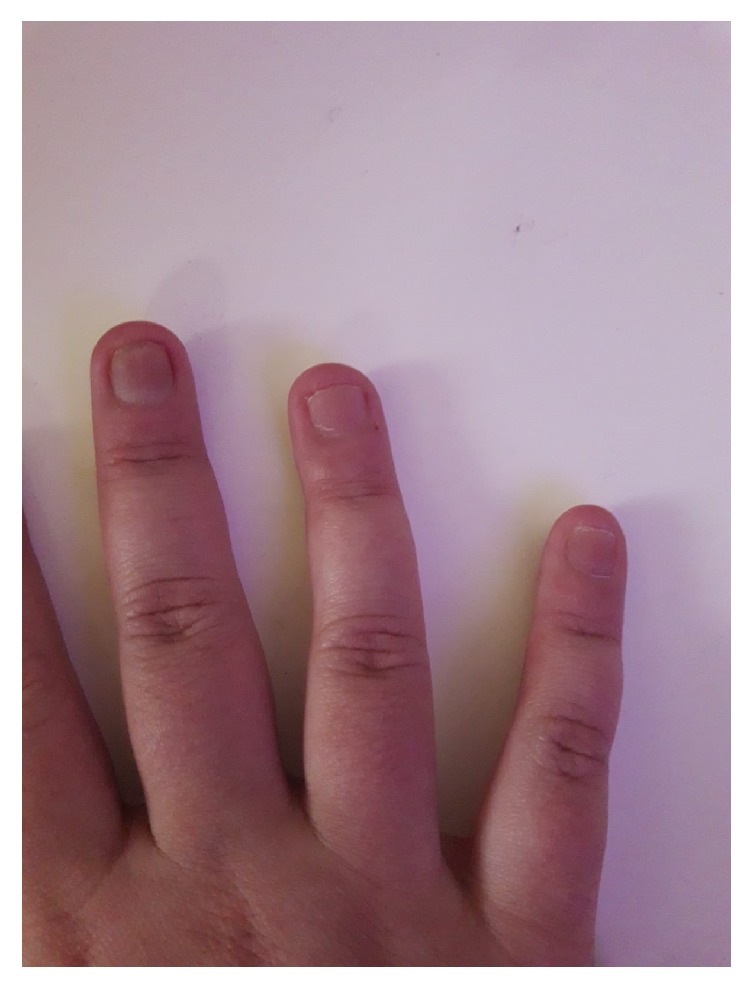
Recovery of nail psoriasis and normal appearance of the nails with a very good response to 22 months of adalimumab therapy.

Biologic treatment was changed to adalimumab 40 mg once every 2 weeks and a full response was achieved for arthritis symptoms after the 3rd injection. She did not experience any articular complaint during 22 months of follow-up. The nail psoriasis on both hands also responded well to adalimumab therapy after few months (Figure 4). Her inflammatory markers regressed to normal values. Treatment-related adverse effects were not detected. She is now well on adalimumab, colchicum dispert (1 gm/day), and subcutaneous methotrexate (20 mg/week) therapy. Clinical and laboratory features of the patient during the disease course were summarized in Table 1.

## 3. Discussion

DIRA (OMIM-612852) was first described in 2009 [2] and its etiology had been linked to loss-of-function mutations in* IL1RN *[1], or a major deletion that involves the* IL1RN* locus [3], a gene that encodes the IL-1 receptor antagonist (IL-1Ra).

Three* IL1RN *mutations, a nonsense mutation, a 2-bp deletion resulting in a frame shift mutation, and a genomic 175-kb, are believed to be founder mutations in their respective populations [9, 10]. In addition, two unrelated Brazilian patients were found to be homozygous for the same 15-bp (in-frame) deletion in* IL1RN, *with a clinical phenotype consistent with the DIRA syndrome [6]. Both patients presented with severe but distinct clinical pictures, mainly involving the skin, bones, and lungs. In 2012, a Turkish patient with a novel Q119∗IL1RN mutation had been reported with an early intrauterine onset and premature death following multiorgan involvement [11]. The mutations observed in our previous case and this case did not lead to an early onset. Our previous case [7] developed pustular cutaneous lesions at one year of age, while our present case presented inflammatory arthritis at 5 years of age. We believe that both of these variants are likely to be typic mutations for late-onset DIRA in the Turkish population.

Conventional disease-modifying antirheumatic drugs such as corticosteroids, methotrexate, and sulfasalazine are of limited benefit, but IL-1 targeted therapies cause dramatic improvement in clinical symptoms and normalize acute-phase reactants [12]. So far, three biologic agents have been approved; anakinra, rilonacept, and canakinumab. Anakinra, a recombinant human IL-1Ra that blocks the proinflammatory effects of IL-1*β*, has been widely used before [4, 12]. Anakinra is given subcutaneously at an initial dose of 1 mg/kg/day and most of the patients with DIRA were reported to have a good response to anakinra treatment. Jesus AA et al. [6] have also reported that their two patients showed a rapid response to treatment with anakinra. The excellent results with anakinra showed that while broad immunosuppression with corticosteroids can result in modest benefit, significant improvement is achieved by increasing competitive inhibition at IL-1R level via recombinant IL-1Ra [5].

However, application of daily subcutaneous anakinra injections is difficult in childhood. A soluble decoy receptor, rilonacept, and another neutralizing human monoclonal anti-IL-1*β* antibody, canakinumab, were also approved. Canakinumab that can be administered every 4-6 weeks began to be a better treatment choice [13]. Our previous patient had responded excellently to canakinumab treatment [7]. The presented case showed partial improvement on canakinumab treatment and then worsened. These two patients, both carrying mutations in* IL1RN*, responded differently to the same treatment. Interestingly, Cowen EW et al. [14] reported that treatment with anakinra in two DIRA patients did not lead to complete normalization of inflammatory markers similarly to what we observed in our patient treated with canakinumab.

Nails are skin appendages. If psoriasis involves the nail matrix, nail psoriasis shows up as changes in the nail plate such as pitting, Beau lines, leukonychia, red spots in the lunula, and nail plate crumbling [15]. Nail psoriasis is accompanied by onychomycosis in up to 27% of cases [14]. Involving the nail matrix or nail bed induces aesthetic problems and functional damage to the patients [16]. Nail psoriasis is less common in children where the prevalence is 7-13%, while in adults it is more common, even in the absence of skin involvement reaching a prevalence of 5-10% [16]. Jesus AA et al. [6] have reported a novel mutation of* IL1RN* presenting with pustular psoriasis and osteomyelitis. Minkis K et al. [10] also have reported IL-1Ra deficiency presenting as infantile pustulosis mimicking infantile pustular psoriasis. Proinflammatory cytokines, such as IL-33, osteopontin, IL-17, and TNF-*α*, are involved in both psoriasis and psoriatic arthritis pathogenesis as well as in bone homeostasis [17]. Recently, IL-36*γ* was also reported to be involved in skin inflammation in psoriasis [18]. Aksentijevich I et al. [2] reported two children with DIRA who had separation of the nail and nail bed similar to the onychomadesis seen in psoriasis. Leung and Lee [19] reported a Hispanic female infant with the clinical, radiologic, and histologic presentation of infantile cortical hyperostosis accompanied with pustular psoriasis. As in some previous cases in the literature, our case had nail psoriasis accompanied with onychomycosis. All dermatologic findings supported the diagnosis.

Nail psoriasis is considered one of the most difficult forms of psoriasis to treat with topical therapies and systemic drugs. Conventional systemic treatments including methotrexate, cyclosporine, and intralesional corticosteroids can be slightly effective for nail psoriasis. The available evidence suggests that all antitumor necrosis factor-alpha and anti-IL17 antibodies are highly effective for nail psoriasis although it is still not clear which is the most effective to treat this particular psoriatic localization [20].

In our case, nail psoriasis and arthritis did not resolve but slightly improved on etanercept and canakinumab therapies. Etanercept has been used for a very long time with improvement at the beginning and unresponsiveness after 7 years. This unresponsiveness to etanercept might be induced by anti-idiotype responses (antibodies against anti-TNF) developed during long term therapy. Besides, a trial of the TNF-inhibitor etanercept, 50 mg twice weekly for 12 weeks and then weekly for another 12 weeks for the treatment of nail psoriasis, showed only 24.2% improvement in nail psoriasis severity index at week 24 (22). In addition, during canakinumab therapy, acute inflammatory reactants did not decrease to normal levels and arthritic symptoms did not fully recover. Similarly, in a study of anakinra for psoriatic arthritis, only 2 of 8 patients with significant skin involvement had improvement [21]. In contrast, skin disease worsened in 4 out of 8, and one patient developed new-onset psoriasis during anakinra treatment [21].

Elewski BE et al. [22] reported a phase 3 trial and evaluated safety and efficacy of adalimumab in patients with moderate-to-severe fingernail psoriasis and moderate-to-severe plaque psoriasis. After 26 weeks of adalimumab treatment, they observed significant improvements in signs and symptoms of moderate-to-severe psoriasis versus placebo and no safety risks were identified [22]. In an open label unblinded study of treatment of nail psoriasis with adalimumab by Rigopoulos D et al. [23], all patients were satisfied while marked improvement in their quality of life was recorded.

The mechanism of adalimumab's positive effects on psoriasis treatment was evaluated by Balato A et al. [24]. Adalimumab treatment downmodulated Th17 responses at skin level [24]. Plasma IL-17 levels and IL-17 positive cells in psoriatic lesional skin were decreased by adalimumab treatment and associated with considerable clinical improvements [24]. Capsoni et al. [25] reported a new and interesting mechanism of action of anti-TNF-alpha mAbs in the control of inflammatory arthritis. Their findings showed that neutrophils are among the targets of anti-TNF-alpha activity [25]. Similarly, Shen C [26] showed that, besides neutralization of TNF-alpha activity, adalimumab induces apoptosis of monocytes/macrophages by activating intracellular caspases and causes inhibition for inflammation. Our previously presented patient did not have an associated problem such as psoriasis. This presented case had both DIRA and psoriasis with onychomycosis symptoms. Therefore, we think that this is the main reason for being well-responsive to adalimumab and not to canakinumab. In addition, the mutations in IL1RN genes of our patients were different which may cause different clinical phenotypes and may give different responses to the same therapies. It is certain that much wide clinical case studies would be needed to formulate a hypotheses of association between different mutations, different phenotypic characteristics, and different therapies.

Our patient's inflammatory arthritis and nail psoriasis with onychomycosis completely recovered on adalimumab therapy. In addition, in the last 20 months her acute phase reactants showing any kind of inflammation had never increased. Her life quality had improved excellently. We can reasonably assume that adalimumab is a valid treatment for psoriatic nails as well as inflammatory arthritis observed in DIRA patients.

In conclusion, if left untreated, the DIRA disease can lead to death from multiple organ failures. If recognized early, the treatment with replacement of the deficient proteins with biologic agents induces rapid and complete remission. This case is remarkable because of her mutation presenting with new clinical phenotypes and responsiveness to a different biologic therapy. This patient is the first one in the literature with the excellent recovery in inflammatory arthritis and nail psoriasis with the monoclonal antibody adalimumab, but not with canakinumab.

## Figures and Tables

**Table 1:** A summary of the clinical and laboratory features during the disease course.

Age (years)	Clinical and laboratory data during disease course

6	She had been diagnosed with bilaterally hand and foot onychomycosis and received anti-fungal therapies with no improvement for about five years

11	Pain, swelling, and limited movement of left elbow, ankles, and both knees and diagnosed with *juvenile idiopathic arthritis* at a public hospital

11	Treatment with corticosteroids, methotrexate and sulfasalazine for one year

12	Admission to Ege University Pediatric Rheumatology Clinic because of not responding to above treatments

12	Limitation in left elbow dorsiflexion, in hip abduction and swelling in both knees, heel pain with tenderness of achilles tendon, dystrophic nails of hand and feet with onychomycosis accompanied with nail psoriasis

12	Very high acute phase reactant levels and hypergammaglobulinemia

12	Diagnosed with *refractory polyarticular juvenile idiopathic arthritis* and *nail psoriasis* with *onychomycosis*. She was started on treatment with *etanercept* (Enbrel), methotrexate and itraconazole with good response for a while for arthritic problems, but not for dermatologic disorders.

13	Because of inadequate response, prednisolone at a dose of 1 mg/kg/day was added and also resulted in substantial improvement

20	No improvement for nail psoriasis with onychomycosis was observed with Etanercept, methotrexate and sulfasalazine therapies for about seven to eight years. In addition, she developed a very severe arthritis in her coxofemoral joint with sacroiliitis and ankylosis.

20	A homozygous premature stop codon mutation c.85C>T (p.Arg29Ter) in *IL1RN gene* was identified and confirmed by Sanger sequencing.

21	Treatment with *canakinumab* 150 mg/4 weeks subcutaneously was given for 9 months and arthritis features slightly recovered, however nail disease did not resolve but slightly improved and acute phase reactants had never decreased to normal levels.

22	Biologic treatment was changed to *adalimumab* 40 mg once every 2 weeks

22	Full response was achieved for arthritis symptoms after the 3rd injection. Her inflammatory markers regressed to normal values.

24	She is now well on adalimumab, colchicum dispert (1 gm/day) and subcutaneous methotrexate (20 mg/week) therapy.
